# On the use of nanomechanical atomic force microscopy to characterise oil-exposed surfaces

**DOI:** 10.1039/c7ra12209h

**Published:** 2018-02-12

**Authors:** Domna-Maria Kaimaki, Ben E. Smith, Colm Durkan

**Affiliations:** Nanoscience Centre, University of Cambridge 11 JJ Thomson Avenue CB3 0FF UK dmk41@cam.ac.uk cd229@cam.ac.uk +44 (0)1223 760 301; BP Exploration Operating Company Limited Chertsey Road Sunbury on Thames Middlesex TW16 7BP UK

## Abstract

Oil-exposed surfaces are susceptible to carbonaceous deposits (CDs). In turn, deposits are responsible for fouling, compromising performance and reducing profitability across the hydrocarbon value chain. An understanding of the deposition behaviour of these organic molecules is therefore imperative. In this paper we address the question of understanding the deposition in upstream operation, where the CDs are known to be asphaltenes, the heaviest fraction of oil. Systematic characterisation of fouled oil-exposed surfaces constitutes an initial step towards that direction and it is a challenging task in itself. We demonstrate the use of Atomic Force Microscopy (AFM) to map surface mechanical properties and how they can be used to determine differences between deposit types. We also demonstrate that the use of an adhesion inhibitor (AI) has a dramatic effect not only on the morphology but also on the mechanical properties of asphaltene deposits.

## Abstract 2

AFM-based maps of elastic modulus superimposed on topography for the case with and without asphaltene inhibitor.

## Graphical abstract

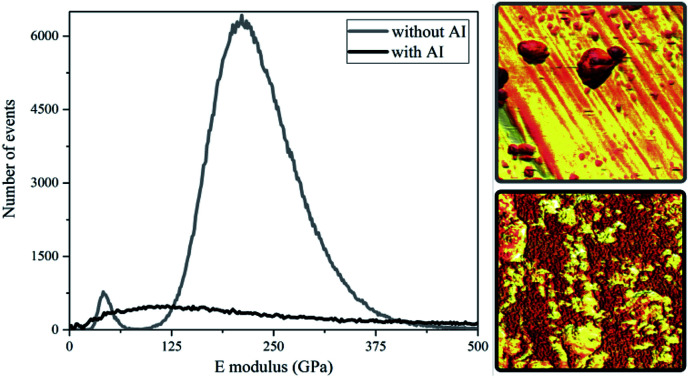

## Introduction

Carbonaceous deposits (CDs) are to be found in all situations where hydrocarbons come in contact with surfaces, be it oil pipelines, refinery components or car engines. In all cases, they lead to a reduction of performance or throughput and are costly to remove. Understanding the nature of these deposits and their interaction with the relevant surfaces is the first step towards being able to mitigate against them. A wide variety of characterisation techniques have been employed in recent years^1–9^ to explore these deposits including electron microscopy and spectroscopy, infra-red spectroscopy, QCM and AFM.

Until now, most literature reports on the use of AFM to investigate oil-exposed surfaces have been limited to the standard modes of AFM imaging – tapping or contact mode.^1,3,5,10–16^ Such imaging and subsequent analysis can generally only provide simple morphological information of the surface including the size of prominent features and the roughness, without any quantitative information regarding the nature of such deposits.

AFM topography and phase imaging of a hard surface is commonplace and of high resolution, subject only to roughness restrictions.^17^ Once asphaltenes or other similar organic soft deposits are found on a surface however, the process of obtaining a stable image can become very challenging which is the reason behind the limited use of AFM to tackle such surface science problems. The deposits are soft and adhesive and thus, they have a propensity to attach to the tip during imaging. Another challenge often faced is that the tip can displace highly mobile molecules from the surface, leading to an inaccurate depiction of the topography.^16^

Nowadays, there is a large variety of advanced AFM modalities, both qualitative and quantitative that can be used to study surfaces. These include measuring and mapping of mechanical properties, electrostatic forces, surface potential, magnetic forces, capillary forces and solvation forces.^18^ Such capabilities, together with the high resolution of the instrument become the comparative advantage of the AFM as a surface characterisation technique and thus, justify reconsidering it to tackle this problem. Moreover, when combined with the spectroscopic capabilities of the techniques mentioned above, useful and meaningful information can be obtained.

In this paper, we report on the use of mechanical property mapping using AFM to explore asphaltene deposits on stainless steel cylindrical rods – referred to as coupons. Asphaltenes are a fraction of oil, which due to their complexity can only be defined as soluble in toluene and insoluble in *n*-alkanes, such as heptane.^19^ They are comprised of polyaromatic rings with aliphatic chains, they are soft, inhomogeneously deposited and often loosely bound to the metal surface, thus presenting a challenge while imaging. One of the first AFM studies depicting asphaltenes was conducted by Toulhoat *et al.*^16^ Even though in his conclusions he recommended that future studies “continue the investigation of the asphaltenes at the molecular scale and focus on understanding the nature of the adhesive forces between the hydrophilic tip and the adsorbed layers of asphaltenes”, over 20 years later, to our knowledge, high resolution AFM images have been limited and the use of nanomechanical AFM modalities has not been reported. Notable exceptions include the article by Lord *et al.*^20^ that presents AFM images of asphaltenes in liquid environments and more recently, the article by Schuler *et al.*^21^ that depicts single asphaltene molecules with atomic resolution. However, this kind of work is neither commonplace nor accessible to most as it requires the use of CO-functionalised AFM tips, UHV and low temperatures.

In our experiments, tapping-mode AFM is used to first take topography and phase images of the sample in an exploratory manner. Once an area of interest is encountered, mechanical property mapping is conducted resulting in maps of the topography, elastic modulus, deformation, stiffness, adhesion force and adhesion energy. Comparisons between (i) different regions of the sample and (ii) samples prepared with and without a proprietary adhesion inhibitor (AI) are carried out.

The aforementioned capabilities of the AFM testify to its versatility, while the results reported here and in other publications,^1,10,16,20,21^ constitute proof of its reliability as a characterisation technique. Hence, the aim of this paper is to provide the reader with a methodology for using tapping mode and nanomechanical mapping mode AFM to examine samples with soft deposits on hard substrates, as well as showcasing key images and data acquired using these modes.

### AFM methodology

There are different modalities used to collect topographic images and a large variety of advanced modalities that can map quantities beyond topography.^18^ The ones used for this research are tapping mode, where the cantilever is oscillated near its fundamental resonance frequency^22^ and HybriD mode (Trademark NT-MDT), where certain mechanical properties of the scan area are derived from the force–distance curve and mapped.

### HybriD mode

Moving beyond topography and phase imaging, the AFM has the capability to do force measurements. The tip–sample force is a function of both the tip–sample separation and the mechanical and chemical properties of the tip and the sample and thus, through the use of an appropriate model, it can be utilised to investigate such properties. This article focuses on the mapping of mechanical properties, with future work moving towards the use of chemical force microscopy in an effort to fully characterise samples by mapping intermolecular potentials and obtaining adhesion maps between chemically functionalised tips and samples.^23–25^ The interaction between two surfaces across a medium is one of the fundamental issues in colloid and surface science. It is not only of fundamental interest but also of direct practical relevance when it comes to dispersing solid particles in a liquid.^26^

**Fig. 1:**
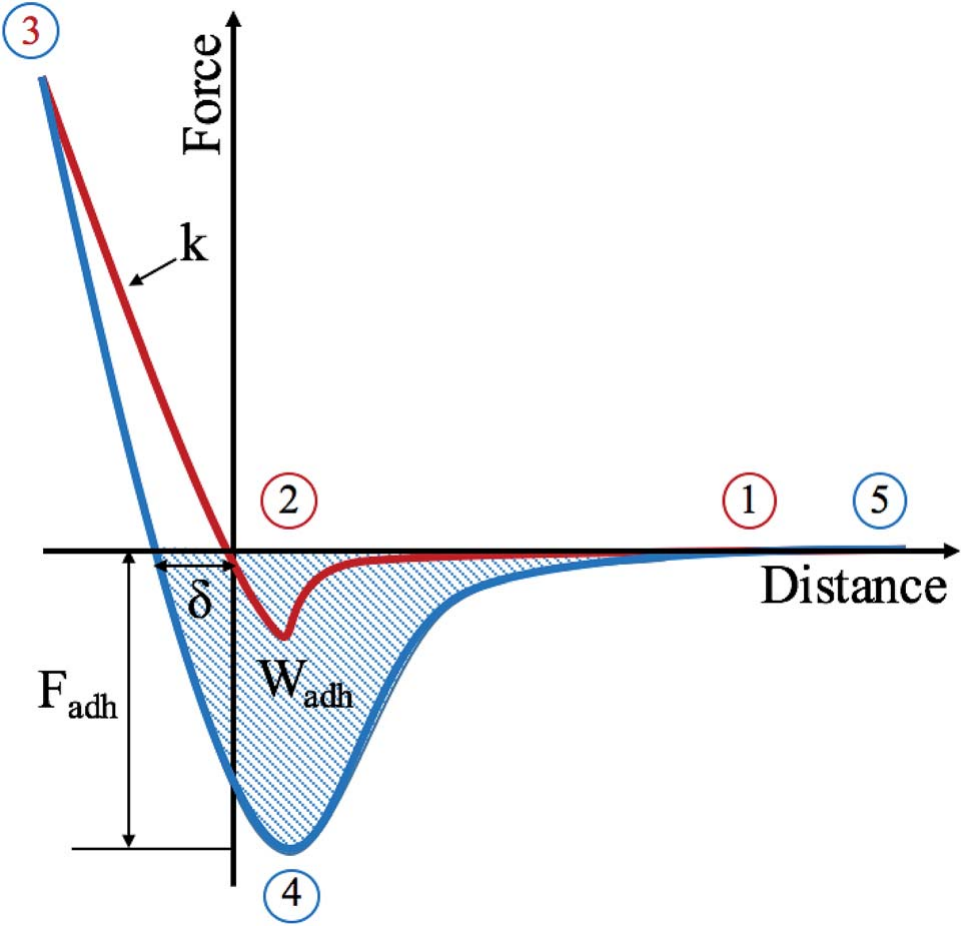
Force curve over an oscillatory cycle where the red line represents the approach and the blue line the retraction of the cantilever from the surface.

In an AFM force measurement the tip is moved towards the sample in the normal direction. During the approach, the cantilever's deflection initially stays at the baseline level shown as point 1 on Fig. 1. As the distance is reduced further, the cantilever starts to bend down due to long-range van der Waals attractive forces (point 2). As the tip snaps into contact, which happens as soon as the force gradient exceeds the cantilever stiffness, short-range repulsive forces start to dominate and as a result the bending reverses upwards until it reaches the set-point level chosen for feedback (point 3). As this process is reversed and the tip is pulled back from the surface, strong adhesive interactions may be experienced by the tip (point 4) until it detaches completely and restores its baseline (point 5). The pull-off force is affected by both long-range and short-range interactions and thus, it is usually larger than the snap-in force, which is governed solely by long-range forces.

For the purposes of the research presented here and in an effort to distinguish between the underlying surface and the deposits, the AFM was operated in HybriD mode to allow for mechanical property mapping. In this mode, the force–distance data obtained is fitted to an appropriate contact mechanics model and mechanical properties are then measured and mapped. The elastic deformation of the sample, *δ*, can be measured from the loading-unloading hysteresis as shown in Fig. 1. The stiffness, *k*, can then be found as it is a measure of the slope of the force curve in the contact part, which can also be used to estimate Young's modulus assuming a certain geometry and material properties of the tip and sample. Two more properties investigated are the force and work of adhesion. The force of adhesion, *F*_adh_, is the force needed for the tip to be able to detach from the surface and return to the baseline and is measured as the difference between point 4 and point 5, as shown in Fig. 1. The work of adhesion, *W*_adh_, is the external work done to separate the adhering surfaces, which is directly related to the surface free energy. It is measured as the area between the baseline and the attractive well created by the retraction part of the curve, as shown in Fig. 1. This is not to be confused with dissipation, which is the amount of mechanical energy lost per tapping cycle. This is measured as the area between the approach and retract curves from the point of peak force (point 3 in Fig. 1) to the point of separation (point 5). The dissipation is therefore generally larger than *W*_adh_.

**Fig. 2:**
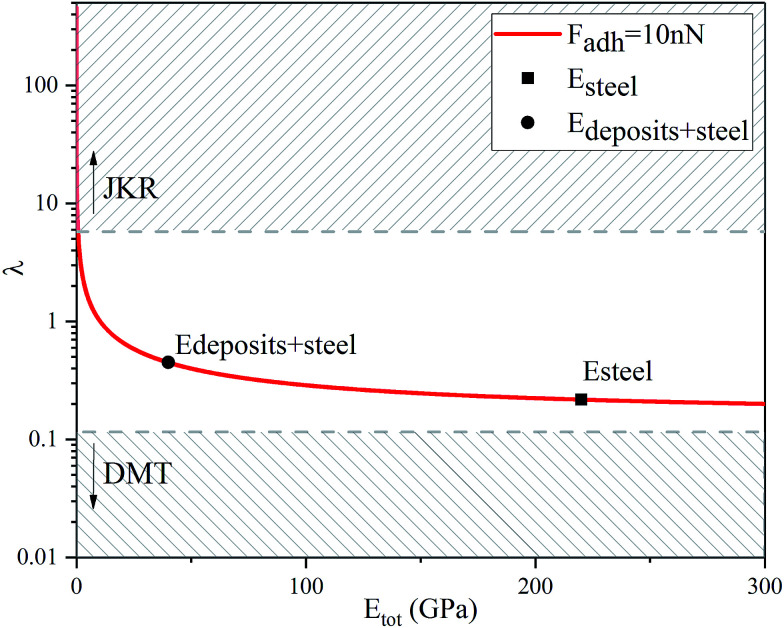
Graphical representation of the Maugis parameter, *λ*, for *F*_adh_ = 10 nN showing that both the JKR and the DMT models are limits of the same theory. The cut-off points for the use of each one of the models are *μ* > 4 for JKR and *μ* < 0.1 for DMT, where *λ* = 1.157*μ*.^31,32^

A key point is that the correct contact mechanics model needs to be chosen for the system under investigation. From the most commonly used models (Hertz,^27^ Derjaguin–Muller–Toporov (DMT)^28^ and Johnson–Kendall–Roberts (JKR)^29^), the DMT and JKR ones are appropriate for the asphaltene–steel system. The Hertz theory neglects adhesive forces and can only be applied when the adhesion force is much smaller than the maximum load, which is not the case for our samples. A typical value for the tip–sample adhesion force that we measure is of the order of 10 nN. It is important to note that both of the appropriate models are only approximations and in fact it was shown by Maugis^30^ that they are limits of the same theory, which mathematically describes the elastic deformations of samples as a function of the parameter
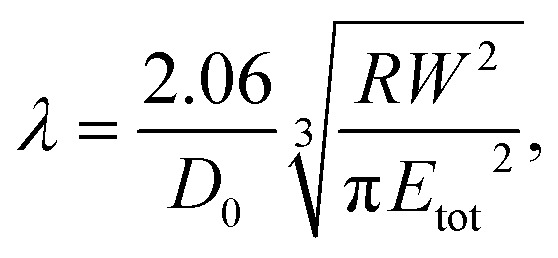
where *D*_0_ is a typical atomic dimension, *R* is the radius of the tip, *W* is the adhesion work per unit area and *E*_tot_ is the reduced Young's modulus accounting for the effect of the tip and surface elastic moduli. A depiction of the Maugis parameter as a function of *E*_tot_ is shown in Fig. 2. The expression is evaluated for an adhesion force of 10 nN, in line with our observations, and the points indicate the *E* modulus of the plain steel surface and that of the steel surface with deposits on top as found experimentally. In addition, there are two lines showing the widely-accepted cut-off points for the use of each one of the models (*μ* > 4 and *μ* < 0.1 for JKR and DMT respectively, where *λ* = 1.157*μ*).^31,32^ This graph highlights the difficulty in choosing the appropriate contact mechanics model initially especially when having limited information on the mechanical properties of the deposits.

It should be noted that the Maugis theory also shows that an exact determination of Young's modulus and work of adhesion only from force–distance curves is not possible since the slope of the contact line and the jump-off from contact point depend on each other. In addition, most contact mechanics theories used for this analysis assume that both the tip and the sample can be described as continuous elastic media and neglect plastic deformations and viscoelastic phenomena.^18^ This is not necessarily true when performing force–distance measurements in relatively soft samples, where viscoelastic effects may take place and manifest themselves as hysteresis between the contact line of the withdrawal curve and the approach contact line as an example. Such effects can be accounted for by control of the amount of indentation and thus, of the resulting stress to the material. However, in the case of very thin soft materials where an indentation of 10% can be unstable, one can account for the presence of inelastic contributions by considering the work of adhesion to be an effective value.^26,33^ These limitations mean that the exact values produced for Young's modulus and work of adhesion are of questionable accuracy, however, mapping the difference in these properties between the surface and the deposits can be a significant result in itself.

Knowing the limitations described above, a choice needed to be made for the most appropriate contact mechanics model for each of the cases we encounter when imaging these samples. The DMT model describes the contact by the Bradley theory,^34^ which considers that all contacting bodies are rigid. Thus, the DMT model is appropriate for surfaces whose elastic modulus is relatively large, surface energy is low and indentation is small (*λ* → 0).^31^ That is the case for our coupons after they have been immersed in toluene and most of the deposits have been removed, so the DMT model for a conical tip geometry was used. However, before immersing the coupons in toluene, they are covered by a thick mat of deposits making their surface softer. In that case, where the material is compliant, the elastic modulus is low, the surface energy is high and the indentation is large (*λ* → ∞), the JKR model for a spherical tip geometry was used. We are confident that this is the appropriate contact mechanics model because the *E* modulus measured is that of the combination of the steel surface with the deposits on top and thus, the *E* modulus of the deposits themselves is expected to be lower and closer to the JKR cut-off line. Moreover, it is shown experimentally that the JKR model can give good predictions even in conditions outside its expected zone.^32^ These differences between the contact mechanics models are summarised in Table 1 shown below.

**Table 1:** Expressions for the contact radius *α*, the sample deformation *δ* and the adhesion force *F*_adh_ for a round tip on a flat surface according to Hertz, DMT and JKR theories. *R* is the tip radius, *W* is the adhesion work per unit area, *F* is the force exerted by the tip on the surface and *E*_tot_ is the reduced Young's modulus defined as 
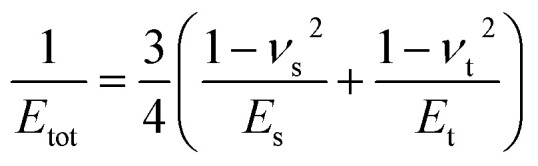
 (ref. 26)

	Hertz	DMT	JKR
*α*	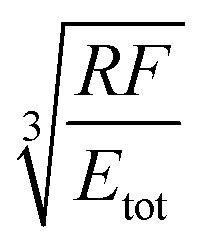	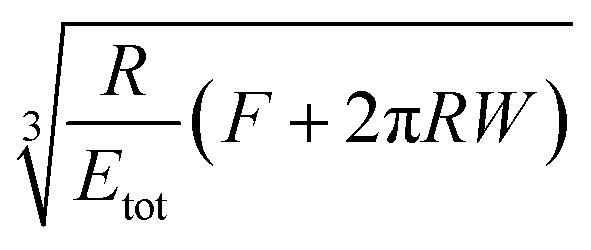	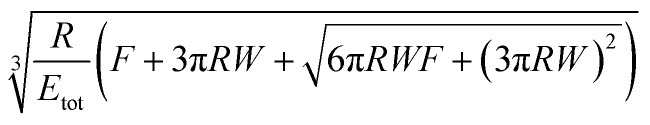
*δ*	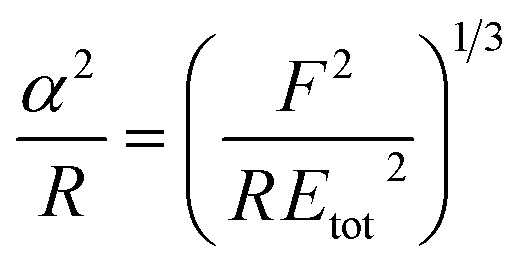	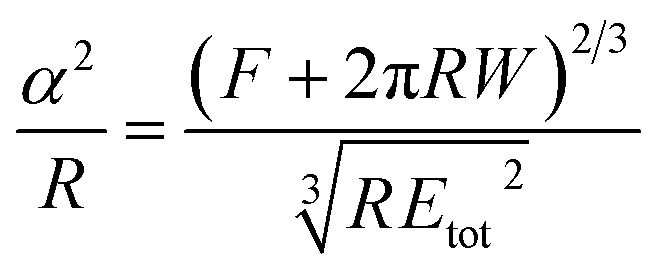	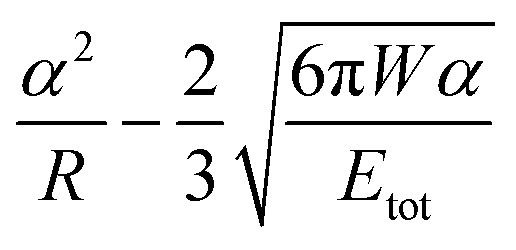
*F*_adh_	0	2π*RW*	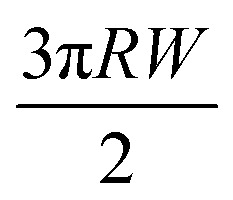

### Optimization

In order to use the aforementioned modalities to successfully image samples with asphaltene deposits on metallic surfaces, there are a number of parameters that need to be chosen, the most important of which is the type of cantilever. Once the AFM cantilever is chosen, the oscillation amplitude, set point, scanning speed and gain of the feedback loop can all be optimised to obtain the best possible results.

### Cantilever

When selecting the stiffness of the cantilever (*k*_c_) it is important to have information on the stiffness of the sample (*k*_s_) and the mode of AFM that is going to be used. If the stiffness of the sample is much higher than that of the cantilever (*k*_s_ ≫ *k*_c_), the cantilever will deflect without any measurable deformation of the sample. This is ideal for topographic imaging since any deformation will result in an underestimation of feature heights. If, on the other hand, the sample is much softer than the cantilever (*k*_s_ ≪ *k*_c_), the cantilever will deform and possibly even indent the sample while scanning.

In our case, the inhomogeneous adsorption of asphaltenes on top of a hard steel surface creates samples that have medium stiffness (Young's modulus of up to tens of GPa). For tapping mode to be used, the cantilever needs to have enough energy to be able to overcome the adhesive forces with the surface. This can be particularly difficult for a cantilever of low stiffness due to adhesion and capillary forces especially for samples with asphaltene deposits. That introduces a minimum limit on how compliant a cantilever can be for this form of imaging. When that is combined with the need for mechanical property mapping, the cantilever needs to bend as well as the tip to induce sample deformation. This suggests that the stiffness of the cantilever used should be comparable to the stiffness of the sample examined (*k*_c_ ≈ *k*_s_). Moreover, when considering tip sharpness, the tips selected were quoted as super sharp in order to be able to obtain good topography images. However, in reality their tip radius was also suitable for mechanical property mapping of medium stiffness materials, as required.

Combining the above information, two cantilevers were chosen for the imaging of asphaltenes on steel surfaces. One had a 325 kHz resonance frequency, stiffness of 40 N m^−1^ (μmasch HQ:NSC15/Al BS) and was used for characterisation of the areas that were mostly metallic without asphaltene deposits. The other one had a 160 kHz resonance frequency and a stiffness of 5 N m^−1^ (μmasch HQ:NSC14/Al BS) and was used for characterisation of the areas where the asphaltenes had adsorbed. Both types had a nominal tip radius of 8 nm and reflective Al coating on the back side.

### Sample preparation

There were two types of samples characterised in this study: 316L stainless steel coupons exposed to crude oil with an asphaltene content of 2.57% both with and without an adhesion inhibitor (AI). The exact chemical composition of the AI is proprietary and thus not known, however, what can be disclosed is that it is a polymer molecule with protic heads and aliphatic tails. The coupons examined here had been immersed in a sealed container of oil with and without AI and stirred for fourteen days. The method of exposure to oil is described in further detail in the literature.^35^

**Fig. 3:**
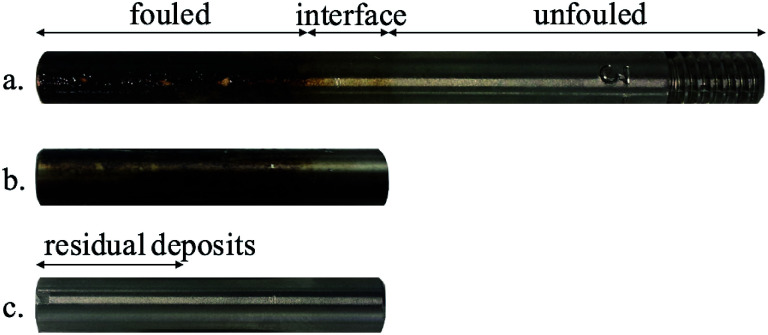
Sample surfaces, referred to as coupons, in the different stages of characterisation: (a) coupon as received, (b) section of coupon and (c) coupon after immersion in toluene.^36^

The CDs on the surfaces analysed were highly inhomogeneous. Using an optical microscope, three different regions could be identified, which were labelled unfouled, interface and fouled according to the amount of deposition found there as shown in Fig. 3. The coupons were initially characterised by Scanning Electron Microscopy (SEM) and AFM with topographical images taken for each of the representative regions. Imaging with SEM proved to be difficult due to charging effects. This could be mitigated by coating the surface, however, that would result in loss of nanomechanical information due to the change in surface chemistry. Thus, it was decided for all the experiments to be conducted using AFM.

Subsequently, 5 cm sections of the coupons were prepared so that they could be better mounted on an AFM for full characterisation, while including areas from all the representative regions. Finally, the coupons were cleaned by immersion in toluene (anhydrous, 99.8%, Sigma-Aldrich) for one minute since asphaltenes are soluble in toluene. The cleaning process was repeated three times until the residual deposits were no longer visible by eye. The coupons were then left to dry in air and the same AFM characterisation process was followed.

Images ranging from a scan size of 45 by 45 μm^2^ to 200 by 200 nm^2^ were acquired for each type of coupon using a XE-100 Park System AFM and a Solver Pro M NT-MDT AFM. In all cases the images were taken under ambient conditions.

## Results and discussion

### Sample as prepared, without AI

**Fig. 4:**
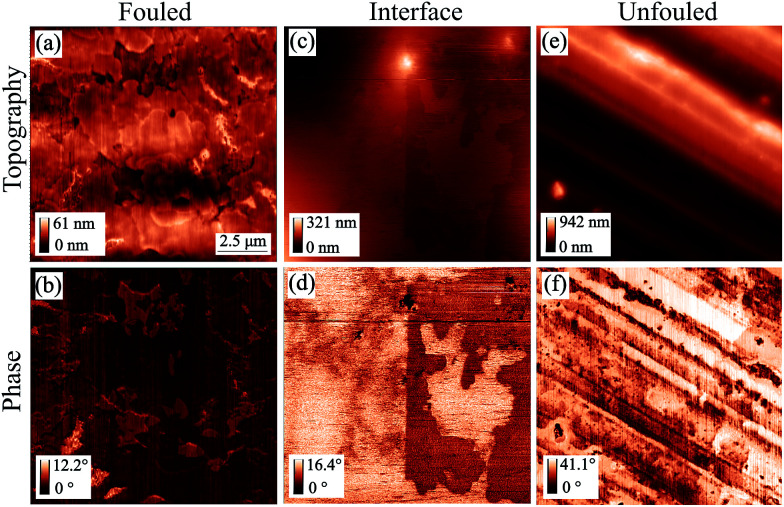
AFM tapping mode topography and phase images of all representative regions of coupon 1 without inhibitor (scan size: 12.5 by 12.5 μm^2^). Images (a) & (b) are from the fouled region, (c) & (d) are from the interface and (e) & (f) are from the unfouled region.^36^

Imaging in the so called unfouled region of the sample revealed its inherent topographic features, which comprise of regular machining grooves 10–100 nm deep along the short axis. Moreover, the phase image showed contrast at the location of the edges of the grooves while it was uniform everywhere else indicating that the material has little variation in mechanical properties across the imaged area, justifying the label assigned to that region (Fig. 4(e) and (f)).

The value of the phase images in regards to asphaltene deposits lies on the fact that there is a substantial difference between the phase shift of the cantilever when it is on a deposit as compared to when it is on a bare metal surface. The polarity of the phase shift can provide information on the interaction between the tip and the sample in that a net attractive force leads to a negative phase shift and a net repulsive force leads to a positive phase shift.^37,38^ Phase imaging is therefore a useful technique to employ when looking at asphaltene deposits even though extracting the sample's material properties in a quantitative manner from such images is not feasible.

The fouled region, shown in Fig. 4(a) and (b), was covered with deposits visible as flakes on the surface. A few distinct features of lateral size around 500 nm to 1 μm and thickness of up to 120 nm have been identified but overall, there is no simple way of determining the thickness of the deposits in this area as they form a continuous mat. This leads to phase images with little contrast as the entire surface is covered with asphaltenes (Fig. 4(b)). The only contrast that does arise is as a result of variations in the mat thickness.

After examining the two extremes of the sample (no coverage to full coverage), the intermediate region, that we are calling the interface, was imaged in an effort to identify an area with thinner deposits. From Fig. 4(c) and (d), a layer of deposits that is around 15 nm thick and appears to be rather uniform can be observed as the bright area in the topography. There was also an indication of clusters as well as nanoaggregates in this region, however, obtaining smaller scan size images of comparable resolution proved to be difficult. The individual clusters were highly mobile and thus, were moved by the tip during the imaging process. It is worth noting that looking at the images of the interface region (Fig. 4(c) and (d)) we would have expected the areas with the higher topography to have a negative phase shift with respect to the 90° phase shift observed at resonance, implying a less repulsive force than the areas of lower topography as is seen for the fouled region for example. However, this is not the case for this region. When operating in tapping mode the cantilever is in the repulsive regime resulting in a positive phase shift relative to when the cantilever is far away from the surface. When imaging soft material on top of metal surfaces the depth of indentation, the relative charge of the different materials and thus the proximity of the tip to the surface are unknown and these conditions can cause the phase inversion that we observe.

**Fig. 5:**
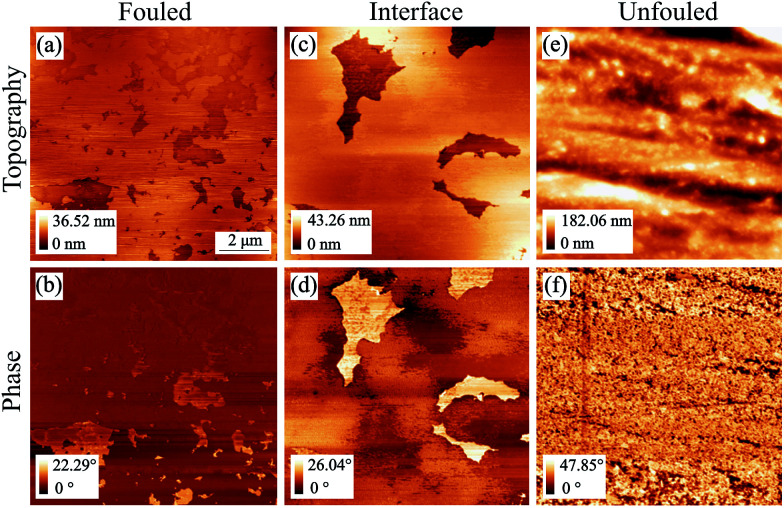
AFM tapping mode topography and phase images of all representative regions of coupon 2 without inhibitor (scan size: 10 by 10 μm^2^). Images (a) & (b) are from the fouled region, (c) & (d) are from the interface and (e) & (f) are from the unfouled region.

When imaging the second sample without inhibitor, shown in Fig. 5, deposits in the form of layers with occasional holes were observed. The deposition behaviour shown in the fouled and unfouled region was similar to that observed in Fig. 4. For this interface region (Fig. 5(c) and (d)), the phase image was consistent with what was originally expected without showing phase inversion.

From the aforementioned characterisation and analysis, we observe that the amount of deposition and the roughness of each of the areas differs. For samples such as these, where there are topographic features covering a large range of length in *x*, *y* and *z* directions, the measured roughness scales with the scan size. Thus, in order to be able to make meaningful comparisons between regions, images of the same scan size should be used.

**Fig. 6:**
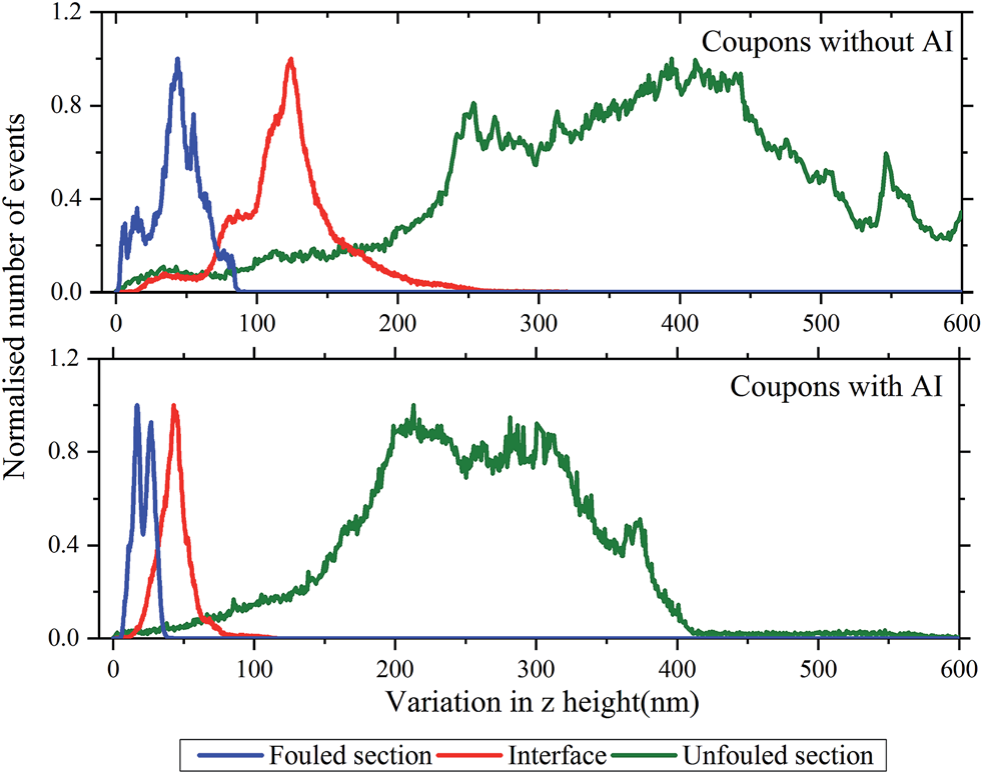
Histograms of the variation in *z* height (*i.e.* surface roughness) in the unfouled (green), interface (red) and fouled (blue) sections of the coupon. The top graph shows the results for coupons without inhibitor and the bottom one the results for coupons with inhibitor.

Surface roughness is quantified by deviations in the direction normal to the surface. Hence in an effort to show the trend in roughness for the different regions, a histogram of the roughness, determined as variations in *z* height with respect to the lowest topography point, is plotted for each of the unfouled, interface and fouled areas as shown in Fig. 6. Observing the histogram of heights corresponding to the unfouled area, a significant variation in roughness can be seen as expected due to the presence of the machining grooves. In the interface region, a decrease in the roughness and its variance is observed showing that the interface is more uniform in *z* height than the unfouled region. This is expected since the high roughness of the coupon's metal surface, shown in the unfouled region, provides nucleation sites for the deposits and promotes adhesion. Thus, the deposits appear to preferentially fill in these grooves. Finally, in the fouled region, there is a further decrease in the roughness and its variance as expected since the deposits have landed everywhere creating a mat and thus, smoothening the underlying surface.

### Sample as prepared, with AI

**Fig. 7:**
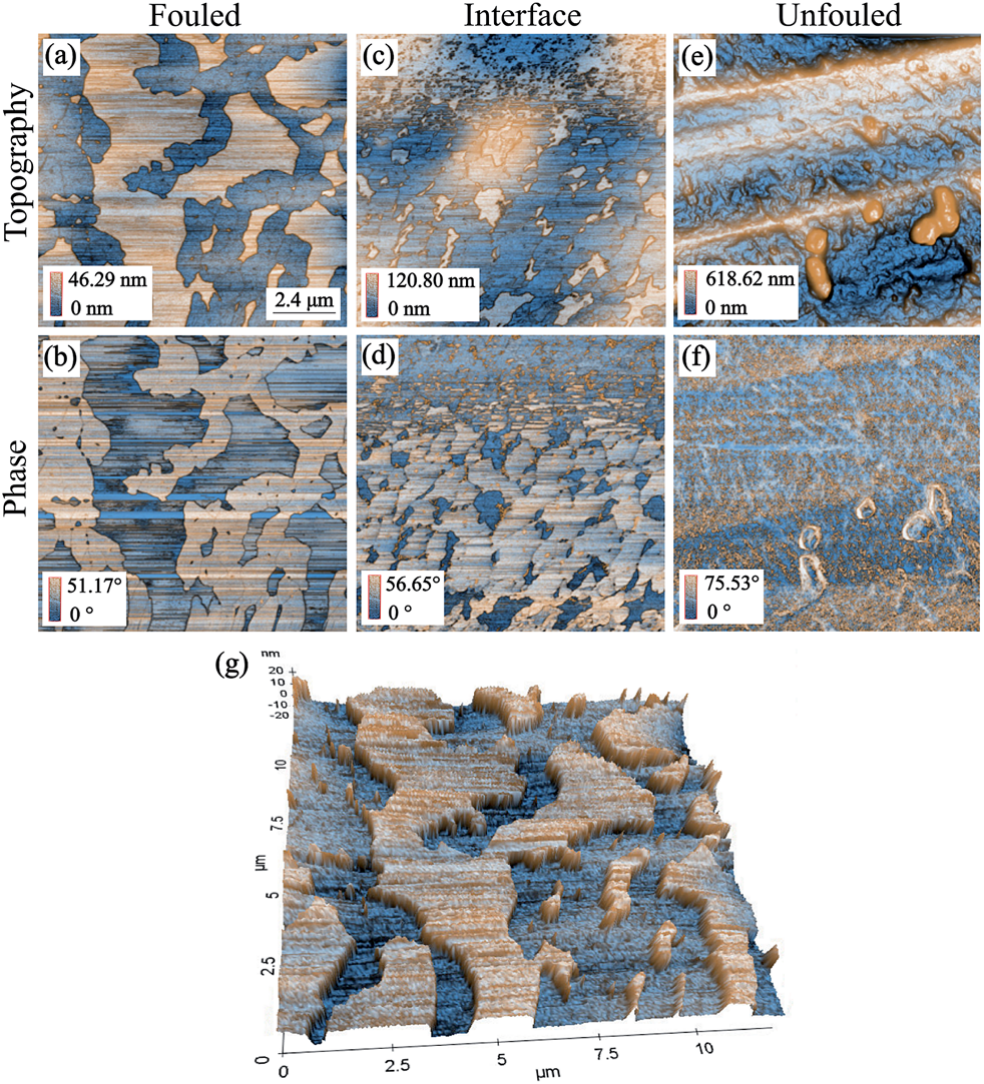
AFM tapping mode topography and phase images of all representative regions of coupon with inhibitor (scan size: 12 by 12 μm^2^). Images (a) & (b) are from the fouled region, (c) & (d) are from the interface region, (e) & (f) are from the unfouled region and (g) is a 3D representation of the topography of the fouled region.^36^

Next, we carried out a similar analysis on samples prepared where AI was added to the crude oil before introduction of the coupons. As with the coupons prepared without AI, imaging in the unfouled region revealed regular machining grooves along the short axis. Moreover, the phase image showed contrast at the location of the edges of the grooves while it was uniform everywhere else indicating that the sample had little variation in mechanical properties across the imaged area as shown in Fig. 7(e) and (f). Some particles were also found on this region, however the phase showed contrast only around the edges of the particles, indicating that they are probably not asphaltene deposits but instead are intrinsic features/defects of the surface.

In the interface region of the coupon, Fig. 7(c) and (d), islands of deposits were observed which display a negative phase shift relative to the 90° phase shift observed at resonance, implying a less repulsive force than the areas of lower topography. There is a big variation in the size of these islands which could imply that some are due to clusters (100 s of nm across, ∼20 nm thick) and some due to nanoaggregates (10 s of nm across and a few nm thick).

**Fig. 8:**
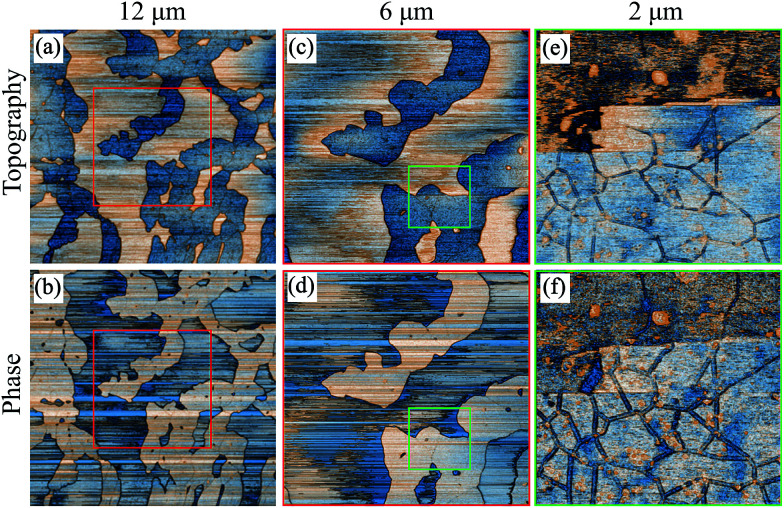
AFM tapping mode topography and phase images focusing on the fouled region of the coupon with inhibitor.^36^

Moving towards the fouled region, Fig. 7(a) and (b) and 3D representation in Fig. 7(g), the deposits are considerably larger. An effort was made to focus further on that area so as to obtain some higher resolution images as shown in Fig. 8. What can be observed in these images is that there are two types of deposit, each with different morphology. The lower regions show deposits that form a compact layer containing drying shrinkage cracks.

This layer could be formed if the deposits were initially physisorbed to the surface and thus, they were quite mobile. If in addition there is a stronger interaction amongst the asphaltenes than between the asphaltenes and the surface, the deposits diffuse together forming a compact layer. The topographic features found on top of that layer comprise deposits that are more mobile as evidenced by the streaks in those areas (which one sees when the tip moves objects as it scans). The fact that they are so weakly bound to the layer of deposits they have adsorbed to indicates that they may be a different species/class of deposits. The separation of the deposits in this way as well as the difference in their adhesion to the surface is clearly an effect due to the inhibitor. Reports from others^39–41^ have shown that the inhibitor will preferentially cover the surface with its protic head attaching to the steel and its aliphatic tail extending outwards. This protective layer of inhibitor makes the binding of the asphaltenes less favourable which explains the decreased adhesion between the surface and the first deposits that adsorb on it that allows for the formation of the initial compact layer of asphaltenes. Such a competitive adsorption mechanism is often investigated by adsorption isotherms or FTIR spectroscopy, however, in this case our knowledge of the AI is limited and thus, this experimental path cannot be followed.^42^ In order to ensure repeatability of the results, a second sample with inhibitor was examined that showed a similar behaviour to the previous coupon.

An analysis of the trends of surface roughness for the different regions was also conducted for the coupon with inhibitor as shown by the variation of *z* height histogram in Fig. 6. The histogram of the unfouled region shows a significant variation in roughness as expected due to the presence of the machining grooves. At the interface, there is a decrease in the roughness and its variance. This is similar to what was observed for the coupon without inhibitor, due to the deposits adhering between the grooves. Finally, in the fouled region, a further decrease in the roughness and its variance is observed. Moreover, in this histogram there are also two distinct peaks corresponding to the compact layer of deposits and those deposits found on top.

**Fig. 9:**
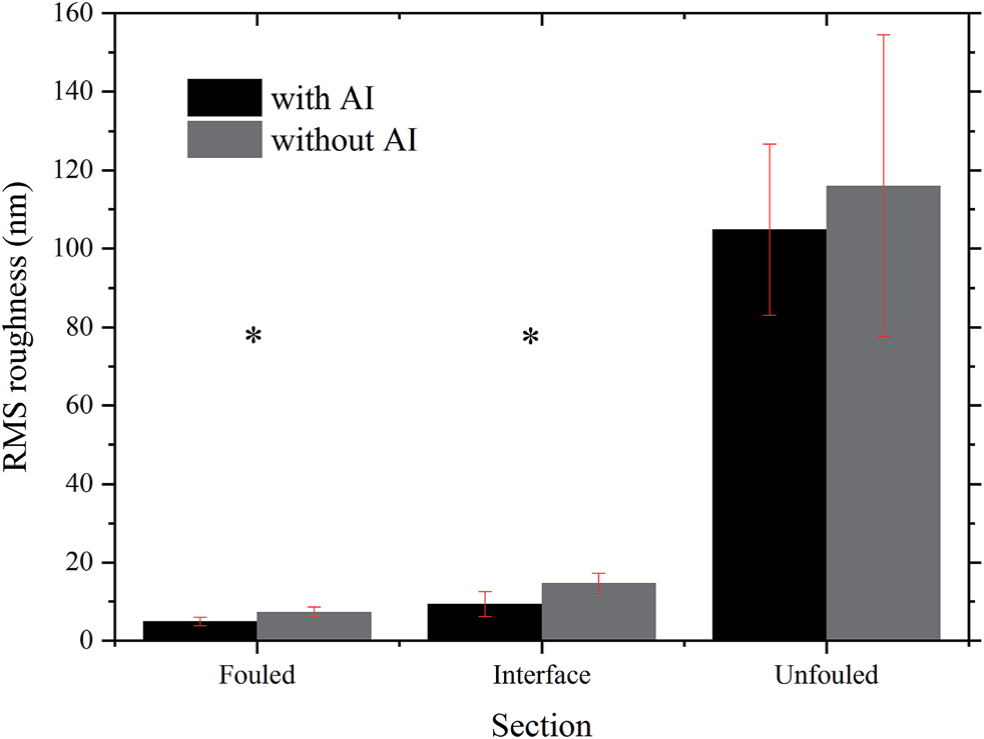
Comparison of the RMS roughness between the different regions of the coupons with and without inhibitor. * indicates the regions whose difference in roughness was found to be statistically significant using a paired *t*-test.

Apart from examining the variation in *z* height between the different regions, the RMS values of each region's roughness on samples with and without AI were also plotted. The bar chart in Fig. 9 shows the RMS roughness and standard deviation based on 5 areas of 2 by 2 μm^2^ scan size, included within the AFM images presented in Fig. 4 and 7. A paired *t*-test was applied to the data which showed that the difference in roughness in the unfouled region is not statistically significant (*p* > 0.05), whereas the difference in roughness between the two types of coupons in the interface and fouled regions is statistically significant (*p* < 0.05). This result confirms that the presence of the inhibitor, found only at the interface and fouled regions, has a measurable effect on the roughness. This is another argument towards the hypothesis that the inhibitor preferentially adheres to the surface. That renders the adsorption of asphaltenes less favourable allowing them to separate and the ones with stronger adhesion amongst them to physisorb and then form layers. Subsequently, the rest of the asphaltenes seem to grow by forming weakly bound clusters on top of the initial layers.

### After rinsing with toluene, without AI

Once the topography of the coupons with deposits already on them was mapped, an effort was made to mechanically characterise them using HybriD mode. However, this proved to be very challenging due to the thickness, adhesive nature and mobility of the deposits. Thus, it was decided to immerse the coupons in toluene, as described in the Sample preparation section, in an effort to remove some of the deposits and characterise the residual ones. This approach was also taken as our interest lies in determining the onset of adsorption and why deposits form where they do.

**Fig. 10:**
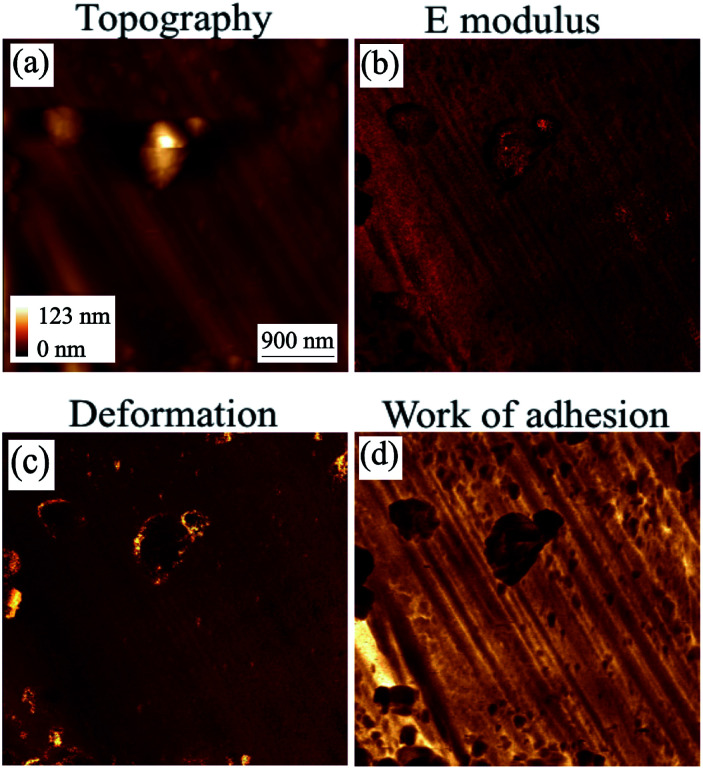
AFM HybriD mode images of the coupon without inhibitor after immersion in toluene. Scan size: 4.5 by 4.5 μm^2^.^42^

Images of scan sizes ranging from 12 by 12 μm^2^ to 2 by 2 μm^2^ were acquired for the region with residual deposits. Fig. 10 shows the results from mechanical property mapping for an area of 4.5 by 4.5 μm^2^. In the topography image (Fig. 10(a)), the regular grooves of the stainless steel surface are observed as well as some deposits. These deposits have a lower elastic modulus than the surface (Fig. 10(b)), as expected, and a higher deformation (Fig. 10(c)) since they are softer. In addition to confirming these expected trends, using the mechanical map of the adhesion energy (Fig. 10(d)) between the AFM tip and underlying surface we were able to observe that the adhesion energy of the steel surface is higher than that of the deposits (|*W*_s_| > |*W*_d_|).

### After rinsing with toluene, with AI

**Fig. 11:**
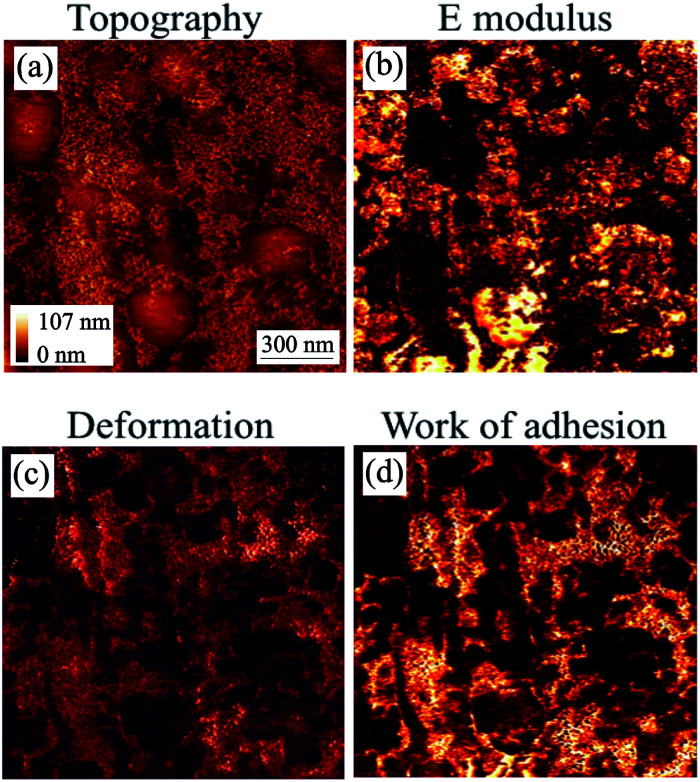
AFM HybriD mode images of the coupon with inhibitor after immersion in toluene. Scan size: 1.5 by 1.5 μm^2^.^36^

Similarly to the coupon without inhibitor, the mechanical properties of the coupon with inhibitor that was immersed in toluene were mapped as shown in Fig. 11. From the topography image (Fig. 11(a)), it is clear that the morphology has been extensively modified by the rinsing process, which removes the most weakly-bound deposits. The smooth features on the topography appear to be the underlying surface, and in between them, there are deposits that are relatively mobile, making stable imaging difficult to obtain. From the HybriD mode mechanical maps simultaneously obtained for the same area, it can be seen that the *E* modulus is lower and the deformation is higher (Fig. 11(b) and (c) respectively) on the deposits as compared to the underlying surface, which is consistent with what is expected. When it comes to adhesion energy (Fig. 11(d)), the adhesion of the surface is shown to be less than that of the deposits. This trend suggests that the underlying surface is not steel but the inhibitor and hence, |*W*_i_| < |*W*_d_| < |*W*_s_|.

**Fig. 12:**
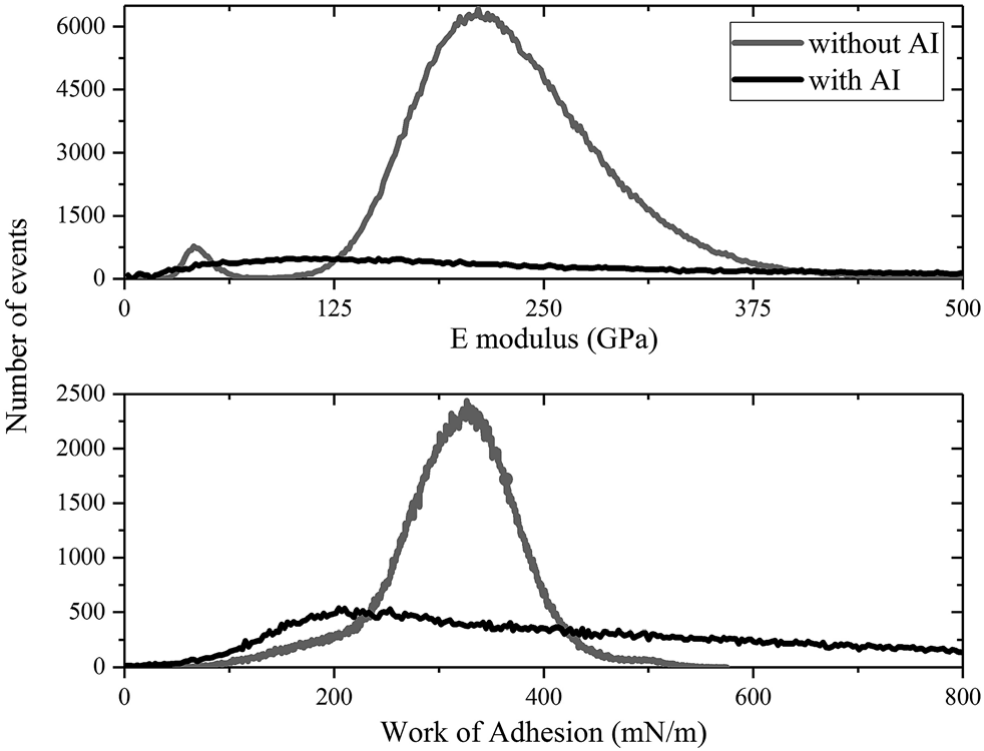
A comparison of histograms of the coupons with and without inhibitor after being immersed in toluene.

Using the maps of the elastic modulus and the adhesion energy provided in Fig. 10 and 11, some further analysis was carried out to obtain their distribution shown in Fig. 12. The top graph of Fig. 12 shows the difference between the *E* modulus of the coupon without AI and that with AI. The *E* modulus of the coupon without AI exhibits two peaks: the bigger one is due to the steel surface (∼220 GPa) and the smaller one is ∼40 GPa, which we can ascribe to the modulus of the deposits observed. This must be taken within the context of the fact that the deposits form a thin (few nm) layer on the steel surface, so this relatively large stiffness value relates to the coupled asphaltene/steel system rather than the asphaltene deposits on their own, the *E* modulus of which would be significantly lower. The *E* modulus of the coupon with AI on the other hand does not show two significant peaks but is significantly broader, consistent with different deposits being present all over the surface. Moreover, the thickness of those deposits is non-uniform and as a result the contribution of the substrate to the measured *E* modulus is highly variable.

The bottom graph of Fig. 12 shows a similar trend where the mean work of adhesion for the coupon without AI is higher than that with AI. Using a grain calculation, we can determine that there is a 10% coverage of the coupon without AI by deposits and thus, the work of adhesion presented is mainly due to the underlying steel. When it comes to the coupon with AI, the same analysis reveals a 69% coverage by deposits, with the rest of the surface covered by an inhibitor. This leads to an expected decrease in the work of adhesion since |*W*_i_| < |*W*_d_| < |*W*_s_|. Moreover, the high variance of the work of adhesion of the coupon with AI is evidence of the different species of deposits with different adhesion found in that case.

Furthermore, a low work of adhesion – between the tip and the surface – as is seen in the coupon with AI means that it is relatively easy for the tip to be separated from the surface. This indicates that the deposits are rather strongly bound to the inhibitor. A comparison of the images in Fig. 10 and 11 confirms the deposits are more difficult to remove in the case of the coupon with AI.

In order to explain the above, we hypothesise that in the case of the coupon immersed in oil with AI, the inhibitor has adhered preferentially to the metal surface. This has rendered the deposition of the asphaltene molecules less favourable and has separated them into different types. One type has adsorbed on the inhibitor surface and due to the adhesion among them being higher than that between them and the inhibitor, they have formed a compact layer. Then another type has deposited on top and has been highly mobile. Once the coupon was immersed in toluene, the mobile deposits were mostly removed; however, breaking the compact layer seemed to be more difficult. More tests need to be performed to confirm this mechanism and compare our experimental results with results from simulations. For example, chemical functionalisation of an AFM tip with some deposit molecules and subsequent nanomechanical mapping or force spectroscopy would allow us to compare the adhesion energy between the steel and the deposits on the tip, |*W*_sd_|, with that amongst the deposits, |*W*_dd_|, and that between the inhibitor and the deposits, |*W*_id_|. Such a measurement conducted in ambient conditions and then in a model oil environment would also allow us to decouple the effect of different types of forces on adhesion.

While more experimental studies need to conducted, our preliminary results suggest that the role of the inhibitor is not to stop deposition altogether but to coat the surface thus making deposition less favourable and to differentiate the deposits. Thus, in the long run the difference between the coupons is expected to be even greater, because the mat of asphaltenes on the coupons without inhibitor is expected to grow significantly. On the other hand, the asphaltenes on top of the compact layer of asphaltenes grown on the coupon with inhibitor are expected to stick less and less, thus not increasing the overall mat of deposits significantly.

## Conclusions

The main observations included in this article are summarised in Table 2 below.

**Table 2:** Summary of observations

	Coupon without AI	Coupon with AI
Before toluene	Topography images of layers of deposits at the interface region and a mat of deposits at the fouled region	Topography images of amorphous deposits on top of a compact layer of deposits
After toluene	Mechanical maps of stainless steel surface with scattered clusters of deposits	Mechanical maps of residual deposits from the compact layer and part of the underlying surface

**Fig. 13:**
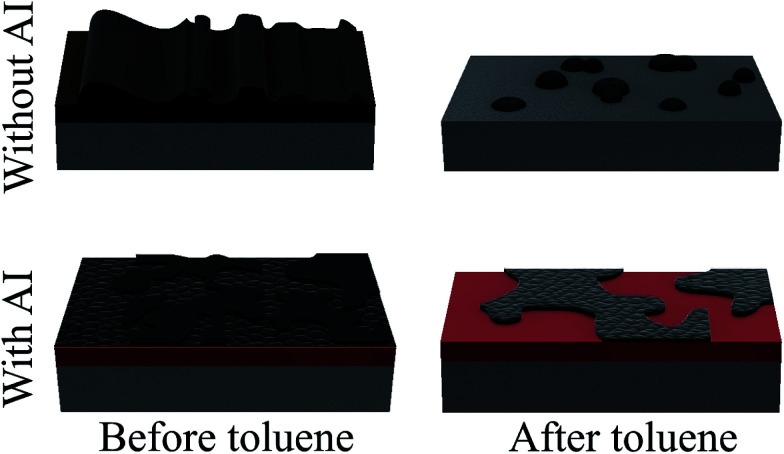
Graphical representation of the deposition mechanism with and without the presence of an inhibitor.

AFM in topography and phase mode imaging has been shown to be a useful tool in the characterisation of asphaltene deposits on metallic surfaces. More specifically, using the aforementioned techniques we were able to show the difference in the nature of deposits with and without an inhibitor. In coupons without inhibitor, highly mobile deposits were observed in the fouled regions and some layers were observed in the interface regions. In coupons with inhibitor, we were able to observe asphaltenes forming a compact layer on the surface as well as weakly bound amorphous deposits on top of them. This difference in the morphology was even more prominent after the coupons were immersed in toluene to investigate the onset of adsorption. In coupons without inhibitor, most deposits dissolved in toluene, thus revealing the steel surface, whereas in coupons with inhibitor, the compact layer of deposits remained as shown in Fig. 13.

However, phase mode imaging is only useful as a qualitative measure of the mechanical properties. Thus, HybriD mode was used to determine the mechanical properties quantitatively and show that even after immersion in toluene the surface is not back to the initial state, *i.e.* the deposition process is irreversible. The data presented shows consistency with what is expected. Further testing and analysis is required however, in order to make any meaningful conclusions on how the residual deposits shift the mechanical properties of the surface.

## Conflicts of interest

There are no conflicts to declare.

## Supplementary Material
